# A new SYBR Green real-time PCR to detect SARS-CoV-2

**DOI:** 10.1038/s41598-021-81245-0

**Published:** 2021-01-26

**Authors:** D. R. Marinowic, G. Zanirati, F. V. F. Rodrigues, M. V. C. Grahl, A. M. Alcará, D. C. Machado, J. C. Da Costa

**Affiliations:** 1 Brain Institute of Rio Grande do Sul (BraIns), Pontifical Catholic University of Rio Grande do Sul, Av Ipiranga, 6690, Building 63, Jardim Botânico, Porto Alegre, Rio Grande do Sul 90610.000 Brazil; 2 Graduate Program in Medicine and Health Sciences, Pontifical Catholic University of Rio Grande do Sul, Porto Alegre, Brazil; 3 Graduate Program in Medicine, Pediatrics and Child Health, Pontifical Catholic University of Rio Grande do Sul, Porto Alegre, Brazil; 4 Graduate Program in Biomedical Gerontology, Pontifical Catholic University of Rio Grande do Sul, Porto Alegre, Brazil; 5 School of Medicine, Pontifical Catholic University of Rio Grande do Sul, Porto Alegre, Brazil

**Keywords:** Molecular biology, Health care, Molecular medicine

## Abstract

Phylogenetic analysis has demonstrated that the etiologic agent of the 2020 pandemic outbreak is a betacoronavirus named SARS-CoV-2. For public health interventions, a diagnostic test with high sensitivity and specificity is required. The gold standard protocol for diagnosis by the Word Health Organization (WHO) is RT-PCR. To detect low viral loads and perform large-scale screening, a low-cost diagnostic test is necessary. Here, we developed a cost-effective test capable of detecting SARS-CoV-2. We validated an auxiliary protocol for molecular diagnosis with the SYBR Green RT-PCR methodology to successfully screen negative cases of SARS-CoV-2. Our results revealed a set of primers with high specificity and no homology with other viruses from the Coronovideae family or human respiratory tract pathogenic viruses, presenting with complementarity only for rhinoviruses/enteroviruses and *Legionella* spp. Optimization of the annealing temperature and polymerization time led to a high specificity in the PCR products. We have developed a more affordable and swift methodology for negative SARS-CoV-2 screening. This methodology can be applied on a large scale to soften panic and economic burden through guidance for isolation strategies.

## Introduction

In December 2019, an outbreak of pneumonia with unknown etiology was identified in Wuhan, China. The outbreak, which likely originated in a seafood market, occurred as a result of zoonotic transmission^1^. In January 2020, the pneumonia outbreak progressed to a nationwide epidemic, which has now become a pandemic. Patients may present with fever, dyspnea, cough, and lung lesions and infiltrates^2^. The clinical picture of patients is similar to that of diseases caused by other coronaviruses, such as the Middle East Respiratory Syndrome (MERS) and Severe Acute Respiratory Syndrome (SARS)^3^. Phylogenetic analyses have proven that the etiologic agent of the Wuhan pneumonia outbreak is a betacoronavirus called SARS-CoV-2^4^. You cannot alter accepted Supplementary Information files except for critical changes to scientific content. If you do resupply any files, please also provide a brief (but complete) list of changes. If these are not considered scientific changes, any altered Supplementary files will not be used, only the originally accepted version will be published.We will not alter the Supplementary Information

The Wuhan pneumonia outbreak has become a pandemic, with confirmed cases on all continents and thousands of deaths around the world^5^. Since we are facing a pathogen for which there is no effective treatment or vaccine, public health measures are necessary to contain the spread of the virus^6^. Isolation and social distancing have been the main tools in the fight to interrupt the chain of viral transmission^7^. For social distancing to be effective, it is necessary to quarantine all individuals who carry the virus. However, some individuals may be asymptomatic, which makes it difficult to diagnose the pathology, and if they are not isolated, they will eventually spread the virus^8^.

To corroborate the effects of public health interventions, laboratory diagnoses of individuals with SARS-CoV-2 is necessary. However, for a proper laboratory diagnosis, techniques with high sensitivity and specificity are necessary, considering that patients may have a low viral load when first infected. Molecular techniques have been designed to address this need^9^. According to the World Health Organization (WHO), the gold standard method to detect SARS-CoV-2 is real-time polymerase chain reaction (RT-PCR) using TaqMan probes, which precisely detect the presence of the virus^10^. However, due to the intensive labor required to perform the technique, the reagents involved, and the limited availability of kits, many diagnoses are based only on late-stage symptoms^11^. Early diagnosis, even when the patient is asymptomatic, is vital to prevent the spread of the virus, as well as for initial prophylaxis with emerging treatments^12^, since the spectrum of this disease in humans is not yet fully understood. Thus, low-cost diagnostic tests must be developed for large-scale patient screening to confirm positive and/or negative cases of the new coronavirus.

To this end, we developed an auxiliary protocol for molecular diagnosis involving RT-PCR with a SYBR Green methodology to detect negative cases of SARS-CoV-2. This protocol will maximize the cost–benefit of viral detection and accelerate availability by the use of conventional kits on a large scale in molecular biology laboratories.

## Results

### Validation of the primer sequences

The Beacon Designer platform showed that there were no secondary structures, no hairpins, no homodimers, and no cross dimer formation in the primer set sequences. Primer-BLAST analysis of both the hCOVassay1 and hCOVassay2 primer sequences showed that they are present only in the target SARS-CoV-2 genome. When base complementarity of the primer set was searched against the human genome, there was no similarity, reinforcing that the viral genome alone would be amplified. In addition, we verified the primers’ ability to anneal in the genomes from other airways circulating opportunistic microorganisms. No relevant homologies were found, i.e., the primers will not generate unspecific amplifications. The searches are shown in Supplementary Table S1 and S2. The primers from hCOVassay1 and hCOVassay2 have proven to be specific only for the SARS-CoV-2 genome.

### RNA extraction and reverse transcription optimization

RNA was extracted using a total RNA extraction kit. The results were below 1 ng/μL according to quantification with the NanoDrop fluorometer. The values of the total RNA extracted were on the pg scale, below the equipment’s detection threshold.

After reverse transcription, the mean amount of ssDNA obtained was 1394 ng/μL (SD = 26.9) for the hCOVassay1 primer, 1327 ng/μL (DP = 107.6) for the hCOVassay2 primer (3′ primer methodology), and 727 ng/μL (SD = 27.3) for the random primers methodology. The ssDNA purity levels were very similar in both techniques. The random primers technique was chosen to produce ssDNA in the first validation stage, since it is easier and quicker than the 3′ primer methodology.

### Template DNA concentration curve for quantitative PCR detection

**Figure 1:**
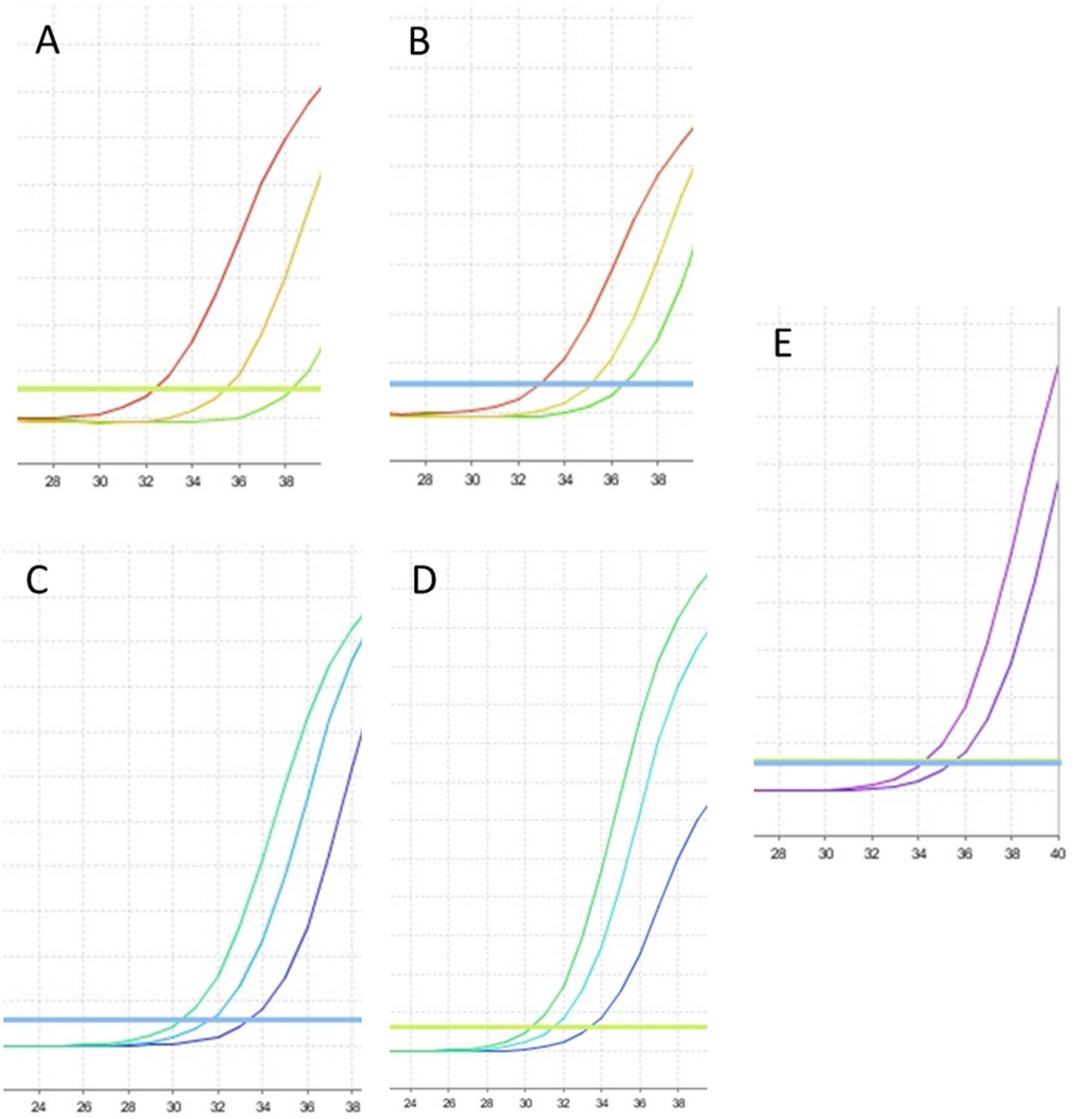
Real-time reverse transcription amplification curves using master mix without uracil DNA glycosylase (UDG) activation. (**A**) Dilutions of ssDNA (500 ng, 100 ng and 50 ng) from SARS-CoV-2 positive samples amplified by the 3′ primer of the hCOVassay1 set. (**B**) Three different dilutions of ssDNA (500 ng, 100 ng and 50 ng) from SARS-CoV-2 positive samples amplified with the 3′ primer of the hCOVassay2 set. (**C**,**D**) The three dilutions of ssDNA (100 ng, 50 ng and 10 ng) from SARS-CoV-2 positive samples produced with the random primers methodology and amplified with the (**C**) hCOVassay1 and (**D**) hCOVassay2 primers. (**E**) Amplification curves of negative controls produced using the two primer sets using 3′ primer ssDNA method. The negative control was from a negative patient.

The manufacturer’s recommended temperatures, time per cycle, and number of cycles were applied to amplify the targeted DNA. The amplifications with the 3′ primer technique and master mix without UDG activation using the hCOVassay1 primer and DNA dilutions of 500 ng, 100 ng and 50 ng yielded cycle threshold (CT) values of 34, 35 and 37, respectively (Fig. 1A). Amplifications with the hCOVassay2 3′ primer generated CT values of 35, 37 and 37, respectively (Fig. 1B). Amplifications of ssDNA reverse transcribed with random primers and master mix without UDG activation using the hCOVassay1 with dilutions of 100 ng, 50 ng and 10 ng yielded CT values of 32, 33 and 36, respectively (Fig. 1C). When the hCOVassay2 primer was used to amplify the same dilutions of the template DNA, the CT values were 33, 34 and 36, respectively (Fig. 1D). The CT value for the negative controls using 3′ primer ssDNA methodology was 34 for the hCOVassay1 primer set and 36 for the hCOVassay2 primer set (Fig. 1E).

**Figure 2:**
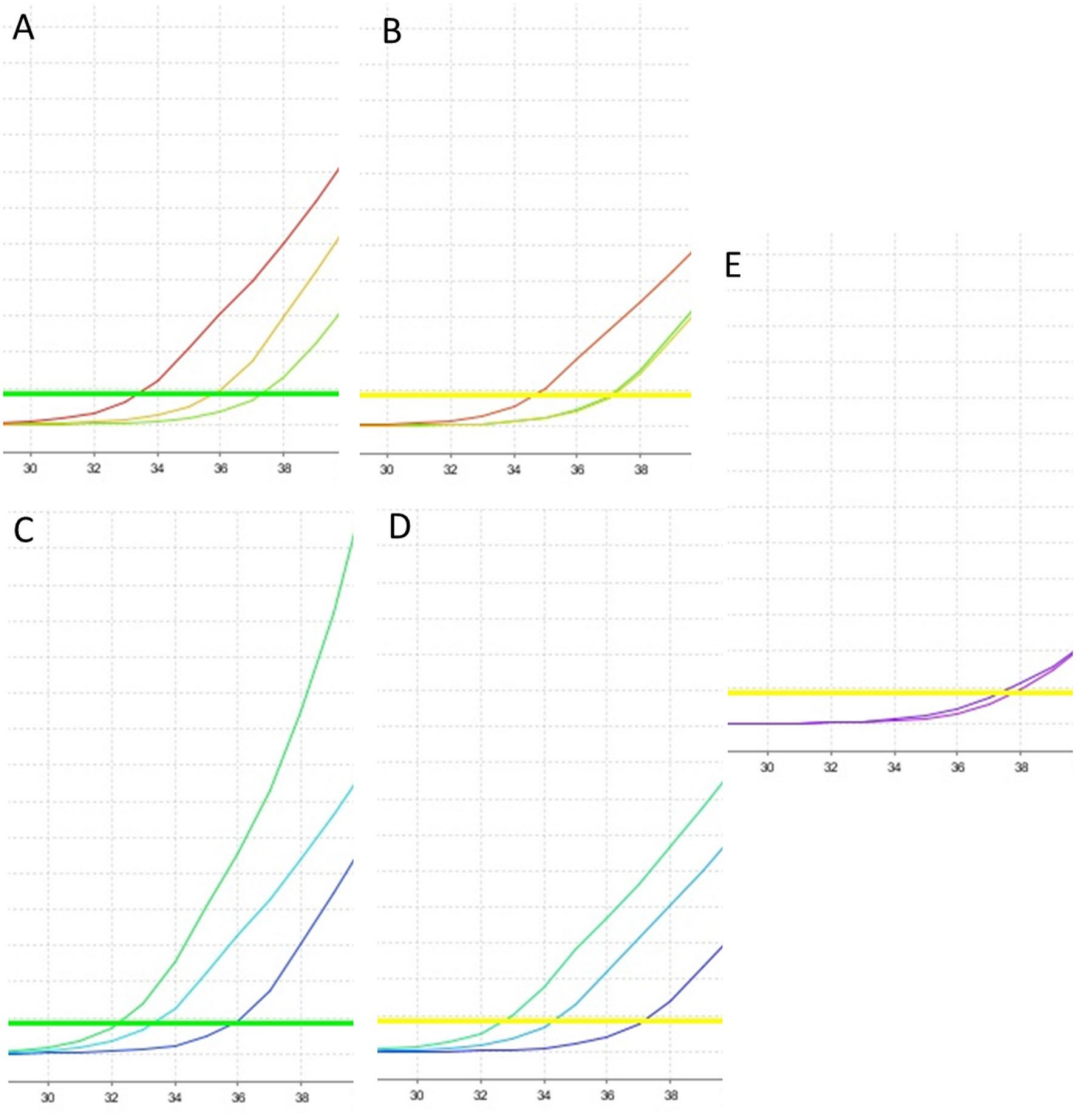
Real-time reverse-transcription amplification curves using master mix with uracil DNA glycosylase (UDG) activation. (**A**) Three dilutions of ssDNA from SARS-CoV-2 positive samples amplified by the 3′ primer of the hCOVassay1 set. (**B**) Three dilutions of ssDNA from SARS-CoV-2 positive samples amplified with the hCOVassay2 primer set. (**C**) Three dilutions of ssDNA from SARS-CoV-2 positive samples produced by the random primers methodology and amplified with the hCOVassay1 and hCOVassay2 primers (**D**). (**E**) Amplified negative controls for the two tested primer pairs. The negative control was from a negative patient.

When master mix with UDG activation was used with the 3′ primer technique using 500 ng, 100 ng and 50 ng dilutions of the template DNA, the CT values yielded for the hCOVassay1 primer set were 34, 34 and 37, respectively (Fig. 2A). The hCOVassay2 primer set generated CT values of 31, 34 and 37, respectively (Fig. 2B). For the reverse transcriptase technique using the random primer methodology and master mix with UDG activation, template DNA with dilutions of 100 ng, 50 ng and 10 ng yielded CT values of 29, 30 and 32, respectively, for the hCOVassay1 primer set (Fig. 2C). When the hCOVassay2 primer set was used, the CT values were 30, 31 and 33, respectively (Fig. 2D). The CT (cut-off) value for the negative controls using 3′ primer ssDNA methodology was 38 for the hCOVassay1 and hCOVassay2 primer set (Fig. 2E).

Thus, the 3′ primer methodology for synthesis of ssDNA was selected as the best option since the parameters of the amplification curves showed less nonspecific signal and a higher level of reliability.

### Melt curve analysis

**Figure 3:**
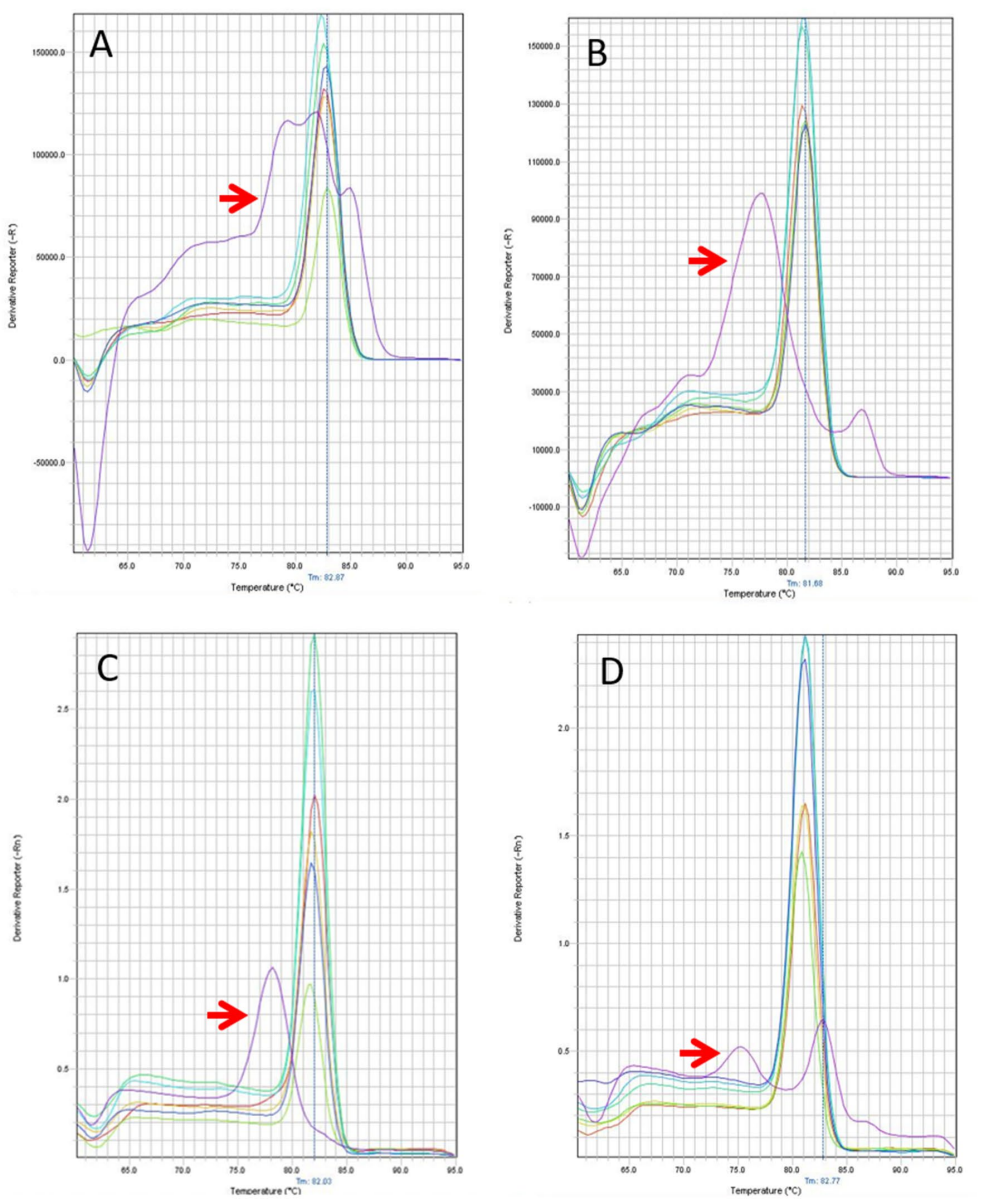
Representation of amplification melt curves after amplification. The melt curves showed a similar pattern for all SARS-CoV-2-positive samples using master mix without UDG activation for the hCOVassay1 (**A**) and hCOVassay2 (**B**) and with UDG activation for hCOVassay1 (**C**) and hCOVassay2 (**D**) primers. The melt curve of the negative samples was quite different from that of the positive samples (arrows). The negative control was from a negative patient.

The melt curve of all amplified SARS-CoV-2-positive samples with both sets of primers produced similar dissociation curves patterns and gave a clear and distinct melt peak (Fig. 3A–D). However, a clearly distinct dissociation curve was obtained for negative control samples that did not align with the melt curve obtained from the positive samples, suggesting that the melt curve peaks generated from the negative samples were due to nonspecific signals or the formation of primer dimers. This parameter is very important in the analysis of specificity of curves for the SyBr Green methodology.

### Visualization of RT-PCR amplicons

**Figure 4:**
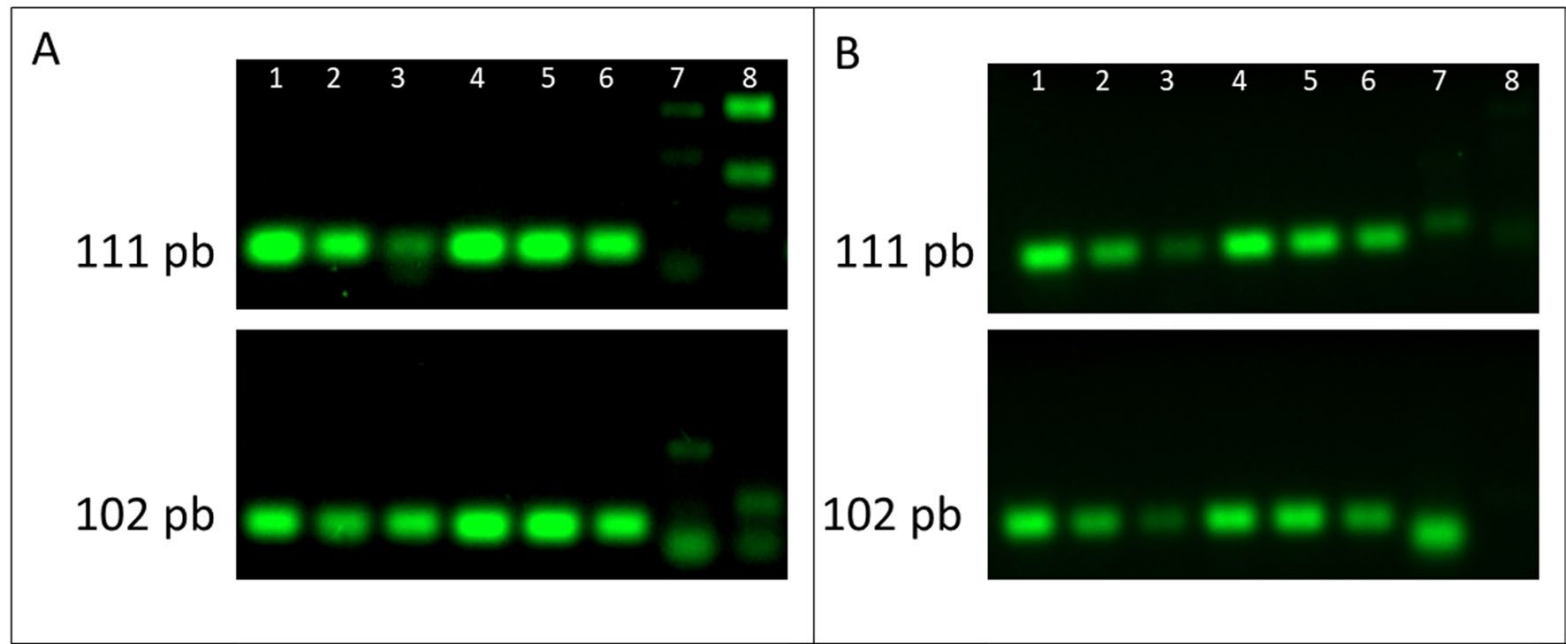
Real-time PCR amplicons of SARS-CoV-2-positive samples after separation by 2% agarose gel electrophoresis. The 111 bp amplicon generated by RT-PCR with the hCOVassay1 primer set (upper figures) and the 102 bp amplicon generated with the hCOVassay2 primer set (lower figures). (**A**) Amplicons produced by RT-PCR without UDG activation. (**B**) Amplicons produced by RT-PCR with UDG activation. Lanes 1–3: 500 ng, 100 ng and 50 ng, respectively, of the first-strand DNA synthesized using the 3′ primer followed by PCR amplification. Lanes 4–6: 100 ng, 50 ng and 10 ng, respectively, of the first-strand DNA followed by RT-PCR amplification. Lanes 7 and 8 are negative controls. The gels were cropped for improving the quality and clarity of image. Full gels are presented in Supplementary Figs. S4 and S5.

The RT-PCR amplicons were separated by electrophoresis, and the products were analyzed under UV to confirm negative and positive samples. Figure 4 shows the 102 bp amplicon corresponding to SARS-CoV-2-positive samples, while the last two lanes show negative samples. When master mix with or without UDG activation was used, the negative control samples did not have a 111 bp band for the hCOVassay1 primer set or a 102 bp band for the hCOVassay2 primer set (Fig. 4: lanes 7 and 8).

### Optimization of amplification parameters

The amplification parameters for each primer were modified (primer annealing temperature and time and Taq DNA polymerization time), which improved the capture signal during the RT-PCR assay, mainly when the master mix with UDG activation was used. The parameter alterations reduced possible nonspecific annealing and hindered the formation of double strands larger than 200 bp.

**Figure 5:**
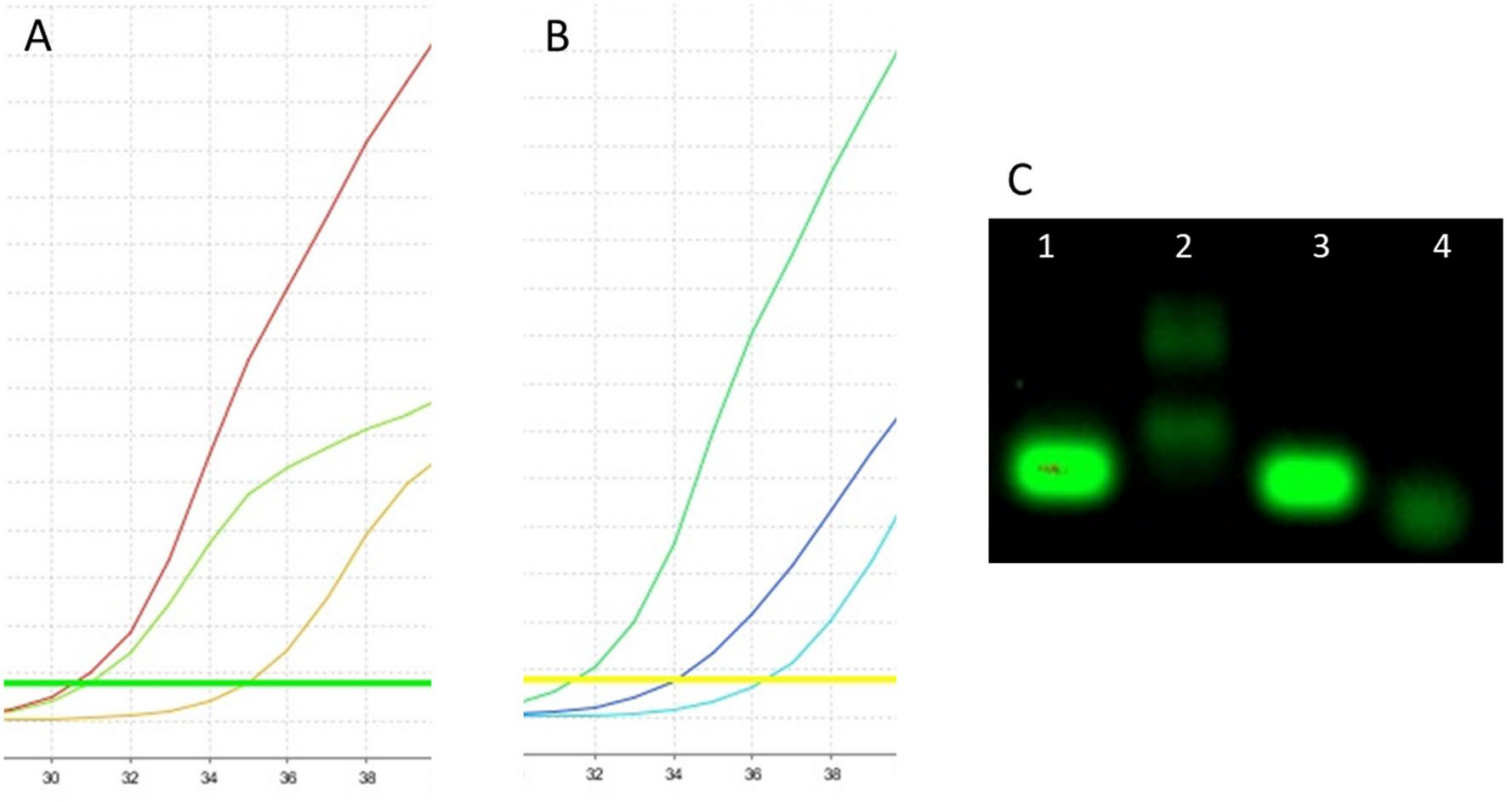
Real-time PCR amplification curves and amplicons of SARS-CoV-2-positive and negative samples without UDG activation followed by separation with 2% agarose gel electrophoresis. (**A**) Amplification curve produced using the hCOVassay1 primer of SARS-CoV-2–positive (red line) and negative control (green and yellow line) samples. (**B**) Amplification curve produced using the hCOVassay2 primer of SARS-CoV-2-positive (green) and negative control (indigo and light blue lines) samples. (**C**) Amplicons after separation on a 2% agarose gel by electrophoresis. Lane 1: amplicon of a SARS-CoV-2-positive sample obtained using the hCOVassay1 primer (111 bp). Lane 2: SARS-CoV-2-negative sample amplified using hCOVassay1 primer. Lane 3: SARS-CoV-2–positive sample amplified using the hCOVassay2 primer (102 bp). Lane 4: SARS-CoV-2-negative sample amplified using the hCOVassay2 primer. The gels were cropped for improving the quality and clarity of image. Full gels are presented in Supplementary Fig. S4.

Figure 5 shows that the RT-PCR amplification curves obtained from the use of master mix without UDG activation produced a faint SYBR signal for the negative controls for SARS-CoV-2 (Fig. 5A,B), although for both primer sets no amplicons can be visualized after separation by electrophoresis (Fig. 5C).

**Figure 6:**
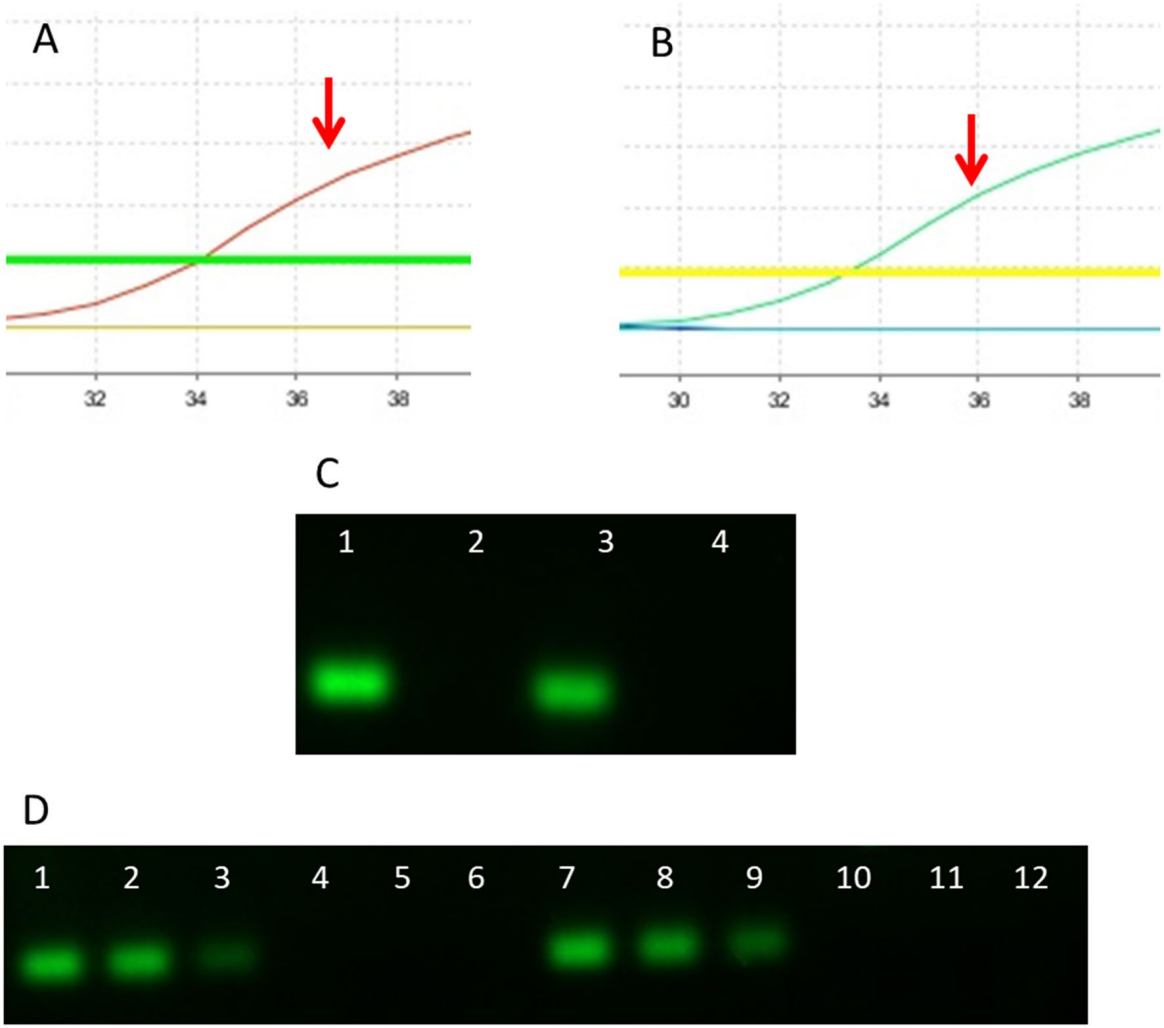
Real-time PCR amplification curves and amplicons of SARS-CoV-2-positive and negative samples with UDG activation followed by separation with 2% agarose gel electrophoresis. (**A**) Amplification curve of SARS-CoV-2-positive samples using the hCOVassay1 primer set (arrow). (**B**) Amplification curve of SARS-CoV-2-positive samples using the hCOVassay2 primer set (arrow). For both primers (**A**,**B**), no amplification or cycle threshold curves were produced for SARS-CoV-2–negative control samples. (**C**) Real-time PCR amplicons. Lane 1: amplicon of a SARS-CoV-2-positive sample using the hCOVassay1 primer set (111 bp). Lane 2: amplicon of a SARS-CoV-2-negative sample using the hCOVassay1 primer set (111 bp). Lane 3: amplicon of a SARS-CoV-2-positive sample using the hCOVassay2 primer (102 bp). Lane 4: amplicon of a SARS-CoV-2-negative sample using the hCOVassay2 primer (102 bp). (**D**) Amplicons of 4 samples and 4 controls for both primers tested in 3 different dilutions of reverse transcriptase produced by the random primers method. Lanes 1, 2 and 3: amplicons of SARS-CoV-2-positive samples using hCOVassay1 primer dilutions of 100 ng, 50 ng, and 10 ng, respectively. Lanes 7, 8 and 9: amplicons of SARS-CoV-2-positive samples using hCOVassay2 primer at dilutions of 100 ng, 50 ng, and 10 ng, respectively. Lanes 4, 5, 6, 10, 11 and 12: amplicons of SARS-CoV-2-negative samples obtained using the two primer pairs. The gels were cropped for improving the quality and clarity of image. Full gels are presented in Supplementary Figs. S5 and S6.

The SYBR Green master mix with UDG activation proved to be very efficient and reliable in terms of non-specific signals. The master mix with UDG activation was more appropriate for avoiding false positives in uninfected human cDNA samples, maintaining the specificity of the amplification signal (Fig. 6).

The Supplementary Table S3 shows the pre-established parameters, as recommended by the manufacturer, and alterations made to improve the detection method are described here.

### Quantifiable control and detection curve

**Figure 7:**
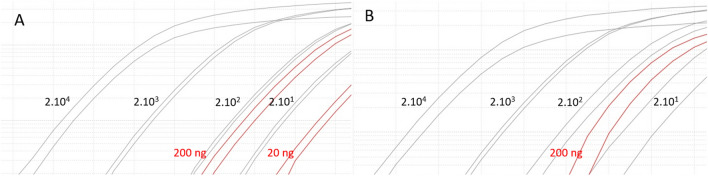
Real-time PCR amplification for quantifiable control and detection curves. Four different concentrations of quantification control for SARS-CoV-2 (iDT—INTEGRATED DNA TECHNOLOGIES, IOWA, USA) using the hCOVassay1 (**A**) and hCOVassay2 (**B**) primer sets are represented in grey curves. The different sample concentrations (total ssDNA after extraction) from positive patient are represented in red curves.

It was possible to detect the presence of the virus by observing the amplification curve from 20 copies of SARS-CoV-2 for both primers. Regarding the detection capacity, less than 20 ng of total ssDNA after transcription using the 3′ primer did not generate a considerably safe amplification curve for virus detection. ssDNA equal to or greater than 200 ng were capable of being detected with great reliability. The comparison of the amplification curves showed that 200 ng of ssDNA corresponds to approximately 200 copies of the SARS-CoV-2 virus in the sample (Fig. 7).

### ssDNA sequencing

**Figure 8:**
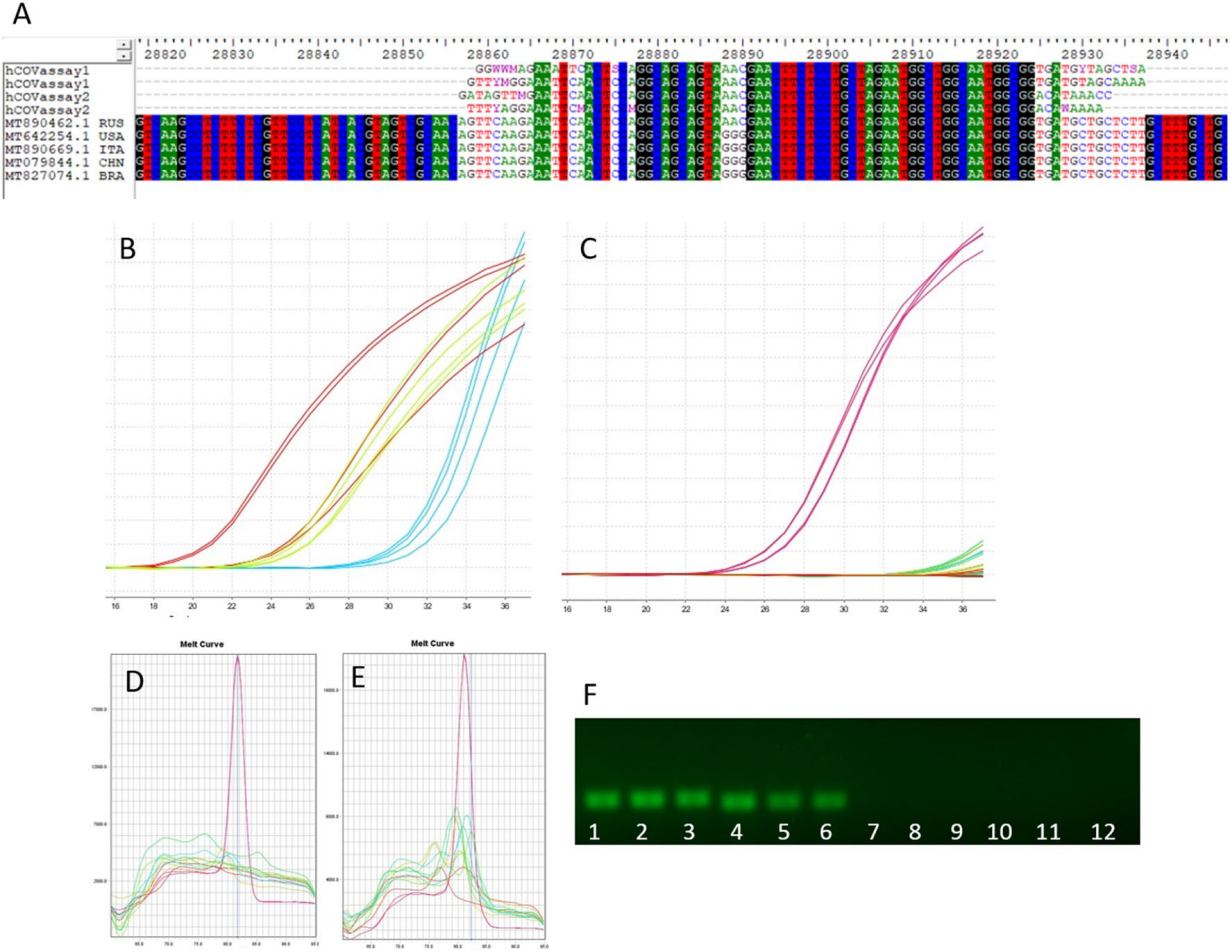
Real-time PCR amplification curves and amplicons of SARS-CoV-2-positive samples with UDG activation using 3′ primer method followed by separation with 2% agarose gel electrophoresis. (**A**) The results obtained by sequencing the amplicons obtained using the hCOVassay1 and hCOVassay2 primer sets showed high homology with SARS-CoV-2 sequences. The homology analysis was performed using entire genome sequences of strain from Russia (MT890462.1 RUS), the United States (MT642254.1 USA), Italy (MT890669.1 ITA), China (MT079844.1 CHN) and Brazil (MT827074.1 BRA). (**B**) Amplification curves for 3 positive samples using the hCOVassay1 and hCOVassay2 primer sets. (**C**) Examples of PCR amplification curves for 6 samples (5 negative samples and 1 positive sample (red curves). Melt curves dissociation for samples amplified using primer hCOVassay1 (**D**) and primer hCOVassay2 (**E**). The melt curve dissociation of the negative and positive samples was clearly distinct. (**F**) Electrophoresis in agarose 2% gel of 3 samples and 3 controls for both primers tested. Lanes 1–3: hCOVassay1 primer. Lanes: 4–6: hCOVassay2 primer. Lanes 7–9 and 10–12: negative controls amplified using the hCOVassay1 and hCOVassay2 primer sets, respectively. The gels were cropped for improving the quality and clarity of image. Full gels are presented in Supplementary Fig. S7.

The amplicons generated through the optimization protocol with SYBR Green master mix with UDG activation were sequenced. For the hCOVassay1 primer, nucleotides showed the highest identity of 100% forward (F) and 95.83% reverse (R) with SARS-CoV-2 from Russia (MT890462.1), 95.45% F and 95.31% R with virus from the United States (MT642254.1), 95.45% F and 95.31% R from an Italian strain (MT890669.1), 95.45% F and 95.31% R from a China strain (MT079844.1) and 95.45% F and 95.31% R from Brazil strain (MT827074.1). The hCOVassay2 showed a similar identity, with 100% SARS-CoV-2 from Russia, 94.92% F and 98.31% R from the United States, 94.92% F and 98.31% R from Italy, 94.92% F and 98.31% R from China and 94.92% F and 98.31% R from Brazilian virus. The alignment of nucleotides after sequencing is shown in Fig. 8A.

### Protocol validation using unknown samples

To validate all variables that improved the distinction between the positive and negative samples, 41 samples from inpatients suspected of COVID-19 infection who were being treated at Hospital São Lucas were used. The samples used in the validation phase were tested with 3′ primers to generate first-strand DNA, RT-PCR performed with master mix with UDG activation and optimized amplification parameters.

Of these 41 samples, 33 were negative and 8 were positive according to this new protocol. All samples were also tested through the CDC protocol for SARS-CoV-2. All 33 negative samples were confirmed, and 7 positive samples were confirmed by the CDC protocol. Only one positive sample according to the SyBr Green protocol was negative in the CDC protocol. The amplification curves of the two set of primers showed a positive signal for confirming positive samples and did not show any signal of amplification curve in negative samples. For confirmation, some samples were submitted for electrophoresis in 2% agarose showing absence of amplicon band. The example of this validation methodology is described in Fig. 8.

The results of the Basic Validation of Qualitative Tests were 100% for positive agreement, 97.1% for negative agreement and 96.7% for overall agreement.

This protocol was applied in approximately 3000 samples. We randomized 300 samples tested from April to September 2020. From these analyzed samples, 48 were positive in SyBr Green and TaqMan protocol and 252 samples were negative in both SyBr Green and TaqMan protocol. A large fraction of samples was tested with the TaqMan protocol only when they were positive for SyBr Green protocol. The examples of this analysis (amplification and dissociation curves) are described in Supplementary Figs. S1 and S2.

## Discussion

The SYBR Green and TaqMan techniques are routinely used in real-time PCR. Due to its simple design, easy configuration and low cost, the SYBR Green detection methodology is predominantly used for the detection and amplification of nucleic acids. However, the TaqMan methodology uses an additional labeled probe, significantly increasing the sensitivity and specificity of the assay due to the reporter dye’s conjugation to the specific oligonucleotide sequence of the probes, which is capable of emitting fluorescence. The TaqMan assay is considered the main method to detect and quantify human pathogens with a low copy number, including viruses^13^.

Nonspecific primer binding, which results in the production of unwanted PCR products and the formation of primer dimers, can significantly influence the sensitivity and reliability of the PCR signals. Therefore, the choice of specific primers and their in silico validation, followed by RT-PCR parameter optimization, plays a fundamental role in the success of an RT-PCR assay when SYBR Green is used.

The hCOVassay1 primer set used in this study, given its complementary to the SARS-CoV-2 sequence, had a high specificity index and generated a 111 bp PCR amplicon. This primer has no homology with the genomic sequences of other viruses from the *Coronaviridae* family or other known human respiratory viruses except rhinoviruses/enteroviruses, which possess a 5 base-pair mismatch with the forward primer and 4 distinct nucleotides on the reverse primer but would generate an amplicon of 409 bp. The forward primer also has five nucleotides homologous to the *Legionella* spp. genome, and the reverse primer has four mismatches, capable of generating an amplicon of 3591 bp. The hCOVassay2 primer set has four and three nucleotides homologous with the *Legionella* spp. genome in the forward primer and the reverse primer, respectively. This set of primers can produce amplicons of 3243 bp and 500 bp, respectively.

The primer annealing temperature and TAQ DNA polymerization time were optimized based on information obtained from the Primer Blast (NCBI) data for each sequence, which allowed an increase in amplification specificity for the PCR products. Annealing temperatures of 54 °C were calculated according to the number of CG/AT bases in each primer pair. In addition, the 20-s polymerization time reduced the possibility of unspecific signals generated by partial primer annealing and/or primer dimers.

The standard WHO methodology uses the TaqMan probe technique to detect SARS-CoV-2. This methodology is extremely effective and can even accurately discriminate between SARS-CoV-2 and SARS-CoV infections. The Charité Protocol, developed at the University of Charité, Berlin, uses four different probes to identify SARS-CoV-2. This test is very expensive, which limits its large-scale application. The U.S. Centers for Disease Control and Prevention (CDC) also has a standardized protocol with four different TaqMan probes that can accurately determine SARS-CoV-2 infection. Both the Charité and the CDC protocols involve reagents and probes that are now scarce and expensive on the world market.

The present study established a screening strategy for SARS-CoV-2 through a molecular assay using the SYBR Green methodology. The proposed method harbors a low cost with the potential for large-scale screening of symptomatic and/or asymptomatic individuals. This screening strategy will allow communities or companies to screen for SARS-CoV-2-negative individuals on a large scale. However, when the test shows a positive result, it is advised that the patient undergo the molecular tests established by the WHO or CDC or even a serological test after the period required to detect antibodies, seven to fourteen days. Therefore, the most scarce and expensive tests could be applied only to a fraction of the target population (Supplementary Fig. S3).

In this study, we established a fast and low-cost method for negative SARS-CoV-2 screening. We suggest that negative by both sets of primers in RT-PCR signal analysis in linear mode can certify the absence of virus. Samples whose signal is compatible with the positive control in one of the two tested primers must be repeated and/or treated as indeterminate. Double-positive samples for the tested primers should be confirmed according to WHO or CDC protocols.

Double-negative patients can resume normality regarding social isolation, while patients who are positive for one or both proposed primers should be immediately tested according to recommended protocols or wait 7 to 14 days for serological tests, also known as rapid tests.

Due to its low cost and processing speed, this methodology can be applied on a large scale, providing peace of mind for those being tested and their peers, as well as guidance for social isolation protocols.

We aimed to develop a low-cost diagnostic test capable of detecting SARS-CoV-2 in the oropharyngeal mucosa of both symptomatic and asymptomatic patients to help reestablish normal work routines as well as to remove SARS-CoV-2-negative individuals from isolation (with appropriate medical follow-up).

## Methods

### Clinical samples

This study was approved by the Research Ethics Committee of Pontifical Catholic University of Rio Grande do Sul (PUCRS) with approval number 3.977.510. All participants provided written informed consent prior to inclusion in this study. All methods were performed in accordance with relevant guidelines and regulations. To determine the specificity of the primer set, the detection curve and possible cross-reactions, a sample obtained from the Clinical Analyses Laboratory of the Hospital de Clínicas de Porto Alegre, RS, Brazil was used. The sample was collected from an inpatient with suspected COVID-19 who attended the São Lucas da PUCRS hospital. The sample was sent to the Laboratório Central de Saúde Pública do Rio Grande do Sul (LACEN) and Fleury Laboratory for determination of SARS-CoV-2 positivity through the Center of Control Disease and Prevention (CDC) protocol.

### In silico study of primer specificity

Two pairs of primers complementary to two different regions of the viral RNA sequence of the isolated coronavirus (Whuan-H1/NC_045512.2) were tested (Supplementary Table S1). First, the primers were input into Beacon Designer Software (PREMIER BIOSOFT INTERNATIONAL) to search their molecular structures based on test type. The SYBR Green option was selected, and the parameters were adjusted accordingly. The primers were then input into the Prime-BLAST platform (NCBI) and evaluated for their specificity with the sequence of the SARS-CoV-2 genetic material. Searches were also performed for similarities within regions of the human genome and of the main respiratory and opportunistic viruses and pathogens to determine whether the primers flanked the regions of interest and amplified only the SARS-CoV-2 genetic material. The input SARS-CoV-2 primers were compared with the genomes of the microorganisms shown in Supplementary Tables S1 and S2.

### RNA extraction and reverse transcription for first-strand DNA synthesis

Nasopharyngeal and oropharyngeal (throat) specimens were collected by a healthcare professional following the CDC instruction guidelines. RNA was extracted from 300 μL of both patient nasopharyngeal and oropharyngeal swab samples 3 to 6 h after sample collection using an SV-Total RNA kit (PROMEGA, MADISON, WISCONSIN, USA). Reverse-transcriptase first-strand DNA synthesis was performed by two methods: (1) a random primer technique using GoScript Reverse Transcription Mix, Random Primers (PROMEGA, MADISON, WISCONSIN, USA), and (2) the 3′ primer technique using M-MLV reverse transcriptase (THERMO FISHER SCIENTIFIC, WALTHAM, MA, USA) with two distinct reverse primers (hCOVassay1 R: 5′AGCAGCATCACCGCCATTG 3′ and hCOVassay2 R: 5′ CCGCCATTGCCAGCCATTC 3′). After the transcription reaction, the product was quantified in a NanoDrop fluorometer (THERMO FISHER SCIENTIFIC, WALTHAM, MA, USA).

### Sample preparation and concentration curve

To determine the specificity and sensitivity of SARS-CoV-2 detection, after reverse transcription, different concentration curves were obtained for the ssDNA of the RNA extracted from SARS-CoV-2-positive samples. The ssDNA was produced through the random primer technique, and amplification was performed using 100 ng, 50 ng and 10 ng dilutions per reaction. First-strand DNA synthesis was performed using the 3′ primer technique, and 500 ng, 100 ng and 50 ng dilutions per reaction were used. Additionally, RNA samples extracted from human skin and brain tissue, previously collected in 2018, were reverse transcribed with random primers for use as a negative control (to ensure potential SARS-CoV-2 negativity).

### Real-time PCR (RT-PCR)

For each reaction, 60 ng of cDNA from the negative controls was used. The samples were amplified using two different master mix compositions, with and without uracil DNA glycosylase (UDG) activation. The GoTaq qPCR Master Mix kit (PROMEGA, MADISON, WISCONSIN, USA) was used for the methodology without UDG activation, while the PowerUp SYBR Green Master Mix kit (THERMO FISHER SCIENTIFIC, WALTHAM, MA, USA) was used for the methodology with UDG activation. The thermal cycles followed manufacturer recommendations and were optimized according to the size of the PCR product and the primer annealing temperature (Supplementary Table S3). Real-time PCR was performed using StepOne Plus equipment (THERMO FISHER SCIENTIFIC, WALTHAM, MA, USA).

### Confirmation of SARS-CoV-2-derived amplicons in 2% agarose gel electrophoresis

The RT-PCR products were separated under electrophoresis in a 2% agarose gel containing ethidium bromide at 100 V for 30 min and analyzed in an automated Gel Doc EZ Gel Imager (BIO-RAD LABORATORIES, INC, CA, USA).

### ssDNA sequencing

Sequencing of samples was performed by ACTGene Análises Moleculares Ltd. (CENTER FOR BIOTECHNOLOGY, UFRGS, PORTO ALEGRE, RS, BRAZIL) using an AB 3500 Genetic Analyzer automatic sequencer equipped with 50 cm capillaries and POP7 polymer (THERMO FISHER SCIENTIFIC, WALTHAM, MA, USA). DNA templates were labeled with 2.5 pmol of the specific primer and 0.5 μL of BigDye Terminator v3.1 Cycle Sequencing Kit (THERMO FISHER SCIENTIFIC, WALTHAM, MA, USA) in a final volume of 10 μL. Labeling reactions were performed in an LGC XP Cycler with an initial denaturing step of 96 °C for 3 min followed by 25 cycles of 96 °C for 10 s, 55 °C for 5 s and 60 °C for 4 min. Labeled samples were purified by 75% isopropanol precipitation followed by 60% ethanol rinsing. Precipitated products were suspended in 10 μL Hi-Di formamide (THERMO FISHER SCIENTIFIC, WALTHAM, MA, USA), denatured at 95 °C for 5 min, ice-cooled for 5 min and electroinjected into the automatic sequencer. Sequencing data were collected using Data Collection 3 software (THERMO FISHER SCIENTIFIC, WALTHAM, MA, USA) programmed with the following parameters: Dye Set “Z”; Mobility File “KB_3500_POP7_BDTv3.mob”; BioLIMS Project “3500_Project1”; Run Module 1 “FastSeq50_POP7_50cm_cfv_100”; and Analysis Module 1 “BC-3500SR_Seq_FASTA.saz”. The resulting Data Collection files (.ab1; electropherograms) were converted into FASTA files (.seq; text) by Sequence Analysis Software v.6 (THERMO FISHER SCIENTIFIC, WALTHAM, MA, USA) with standard parameters.

### Dilution and detection curve

A dilution curve was performed using quantifiable SARS-CoV-2 Control (iDT—INTEGRATED DNA TECHNOLOGIES, IOWA, USA). Serial dilutions of 20,000, 2000, 200, 20 and 2 copies of the virus were tested. To establish detection capacity, serial dilutions of the ssDNA of a positive sample for SARS-CoV-2 were performed at the following concentrations: 200 ng, 20 ng, 2 ng and 0.2 ng.

### Protocol validation

After establishing the best concentration criteria and methodology for diagnosis, a validation step was performed with samples from 15 patients with suspected SARS-CoV-2 infection treated at Hospital São Lucas. Reverse transcriptase using random primers followed by the RT-PCR methodology with UDG activation and sample concentrations above 100 ng per reaction (Supplementary Table S3) were applied to confirm the specificity of this new method. The protocol was validated using a Basic Validation of Qualitative Tests through WestgardQC software (https://www.westgard.com/validating-qualitative-tests.htm).

## Supplementary Information

Supplementary Information.

## Data Availability

The datasets generated and/or analyzed during the current study are available from the corresponding author on reasonable request.
