# Supplementary material for: A new SYBR Green real-time PCR to detect SARS-CoV-2
Source: Sci Rep. 2021 Jan 26;11:2224. doi: 10.1038/s41598-021-81245-0 (PMC7838253; doi:10.1038/s41598-021-81245-0)
Supplement: Supplementary file 1 — Supplementary Information. [file 41598_2021_81245_MOESM1_ESM.docx]

**A new SYBR Green real-time PCR to detect SARS-CoV-2**

Marinowic, D.R.^a,b,c,d^., Zanirati, G.^a,c^, Rodrigues, F.V.F.^a,c^, Grahl, M.V.C.^a,b^, Alcará, A.M.^a, c^, Machado, D.C.^a,b,d^, Da Costa, J.C.^a,b,c*^

**Supplementary Table S1**. hCOVassay1 primer set complementarity data using PrimerBlast software

*hCOVassay1 primer*

*Forward*: 5’GCCTCTTCTCGTTCCTCATCAC 3’

*Reverse*: 5’AGCAGCATCACCGCCATTG 3’

| Target organism | Amplicon (bp) | Access code (RefSeq) | Forward template | Reverse template | Mismatch | Taxonomy ID |
| --- | --- | --- | --- | --- | --- | --- |
| *SARS-CoV-2* | 111 | MT226610.1 | 28817-28838 | 28927-28909 | W/C | 2697049 |
| *Rhinovirus/*  *enterovirus* | 406 | MK989760.1 | 5822-5801 | 5417-5435 | Forward: 2 C/G; 3C/A; 5C/G; 16T/A;18A/T  Reverse: 1A/G; 5G/A; 13G/C; 14C/T; 17T/G | 12059 |
| *Rhinovirus/*  *enterovirus* | 406 | MK989750.1 | 5464-5443 | 5059-5077 | Forward: 2C/G; 3C/A; 5C/T; 16T/A; 18A/T  Reverse: 1A/G; 5G/A; 13G/C; 14C/T; 17T/G | 12059 |
| *Legionella spp.* | 3591 | LR134380.1 | 1098252-1098231 | 1094662-1094680 | Forward: 12T/C; 13T/C; 16T/A; 17C/T; 20C/T  Reverse: 2G/C; 3C/G; 11C/A; 14C/T | 445 |
| *Legionella spp.* | 3591 | LS483412.1 | 1098250-1098229 | 1094660-1094678 | Forward: 12T/C; 13T/C; 16T/A; 17C/T; 20C/T  Reverse: 2G/C; 3C/G; 11C/A;14C/T | 446 |

bp= base pairs

**Supplementary Table S2**. hCOVassay2 primer set complementarity data using PrimerBlast software.

*hCOVassay2 primer*

Forward: 5’ AGCCTCTTCTCGTTCCTCATCAC 3’

Reverse: 5’ CCGCCATTGCCAGCCATTC 3’

| Target organism | Amplicon (bp) | Access code  (RefSeq) | Forward template | Reverse template | Mismatch | Taxinomy ID |
| --- | --- | --- | --- | --- | --- | --- |
| *SARS-CoV-2* | 102 | MT226610.1 | 28816-28838 | 28917-28899 | W/C | 2697049 |
| *Legionella spp*. | 3243 | LR134173.1 | 3094031-3094013 | 3090789-3090807 | Forward: 3G/T; 7T/C; 10C/G; 19C/T  Reverse: 3G/C; 13G/A; 17T/C | 445 |
| *Legionella spp.* | 500 | CP038271.1 | 187989-187971 | 187490-187508 | Forward: 2C/G; 10C/T; 12A/C; 18T/G  Reverse: 1C/G; 5C/T; 6A/C; 9G/T | 445 |

Organisms investigated (Taxonomy ID) but with no complementarity where as follows: *HCoV-HKU1* (290028); *HCoV-OC43* (31631); *HCoV-NL63* (277944); *HCoV-229E* (11137); *MERS-CoV* (1335626); *H1N1* (114727); *H3N2* (119210); *Influenza* (untyped) (11309); *H5N1* (102793); *H7N9* (333278); *Influenza B* (11520); *Respiratory Syncytial Virus* (11250); *Parainfluenza 1 virus* (12730); *Parainfluenza 2 virus* (1979160); *Parainfluenza 3 virus* (11216); *Parainfluenza 4 virus A* (11224); *Parainfluenza 4 virus B* (11226); *Human Metapneumovirus* (162145); *Human Bocavirus* (329641); *Mycoplasma spp.* (2093). *Rhinovirus/Enterovirus* (12059) no complementarity was found with the primer set two.

**Supplementary Table S3**. Real-time PCR conditions adapted for the use of SARS-CoVassay-1 and SARS-CoVassay-2 primer sets with or without UDG activation in the reaction master mix.

| + UDG activation   \| Temp \| Time (min) \| Cycles \| \| --- \| --- \| --- \| \| 50°C \| 2:00 \| hold \| \| 95°C \| 2:00 \| hold \| \| 95°C \| 0:15 \| 40 \| \| 60°C \| 1:00 \|  \| Temp. \| Time (min) \| Cycles \| \| --- \| --- \| --- \| \| 50°C \| 2:00 \| hold \| \| 95°C \| 2:00 \| hold \| \| 95°C \| 0:15 \| 40 \| \| 54°C \| 0:20 \| \| 72°C \| 0:20 \|   Reference  Adapted PCR conditions | no UDG activation   \| Temp. \| Time (min) \| Cycles \| \| --- \| --- \| --- \| \| 95°C \| 2:00 \| hold \| \| 95°C \| 0:15 \| 40 \| \| 60°C \| 1:00 \|  \| Temp \| Time (min) \| Cycles \| \| --- \| --- \| --- \| \| 95°C \| 2 minutes \| hold \| \| 95°C \| 0:15 \| 40 \| \| 54°C \| 0:20 \| \| 72°C \| 0:20 \|   Reference  Adapted PCR conditions |
| --- | --- | --- | --- | --- | --- | --- | --- | --- | --- | --- | --- | --- | --- | --- | --- | --- | --- | --- | --- | --- | --- | --- | --- | --- | --- | --- | --- | --- | --- | --- | --- | --- | --- | --- | --- | --- | --- | --- | --- | --- | --- | --- | --- | --- | --- | --- | --- | --- | --- | --- | --- | --- | --- | --- | --- |


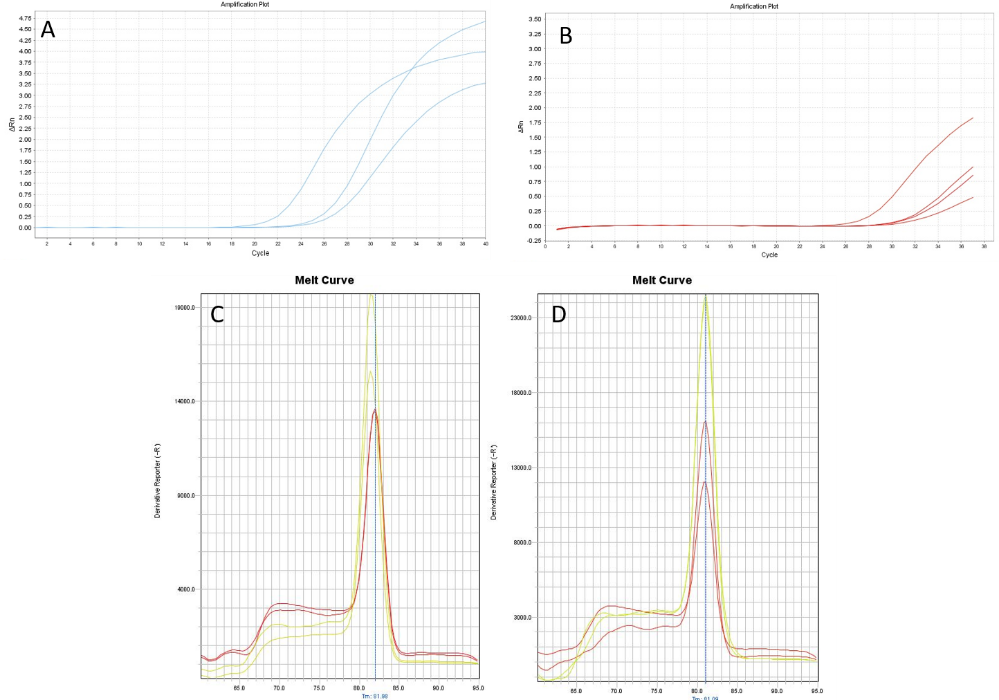


**Supplementary Figure S1.** Real-time PCR amplification curves of SARS-CoV-2 positive sample. A) Amplification curve produced using the TaqMan probe technique to detect SARS-CoV-2 (N, E and RdRp gene). B) Amplification curve produced using the hCOVassay1 and hCOVassay2 primer set. The Melt curves dissociation for sample and control amplified using primer hCOVassay1 (C) and primer hCOVassay2 (D).


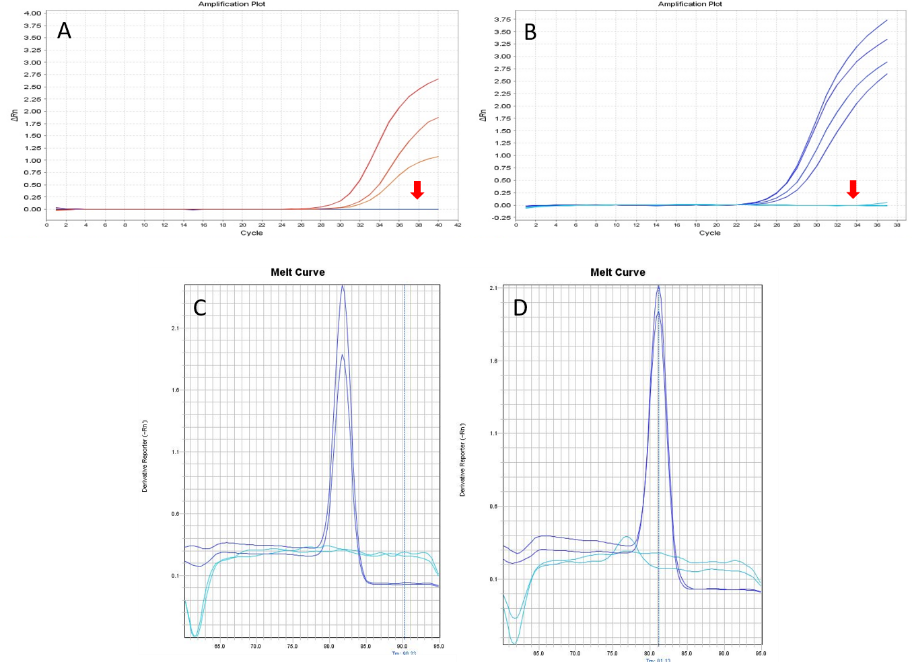


**Supplementary Figure S2.** Real-time PCR amplification curves of SARS-CoV-2 negative sample. A) Amplification signal produced using the TaqMan probe technique to detect SARS-CoV-2 in negative sample (red arrow) and positive control (red curves). B) Amplification signal produced using the hCOVassay1 and hCOVassay2 primer set in negative sample (red arrow) and positive control (dark blue) . The Melt curves dissociation for negative sample (clear blue) and positive sample control (dark blue) amplified using primer hCOVassay1 and (C) and primer hCOVassay2 (D).


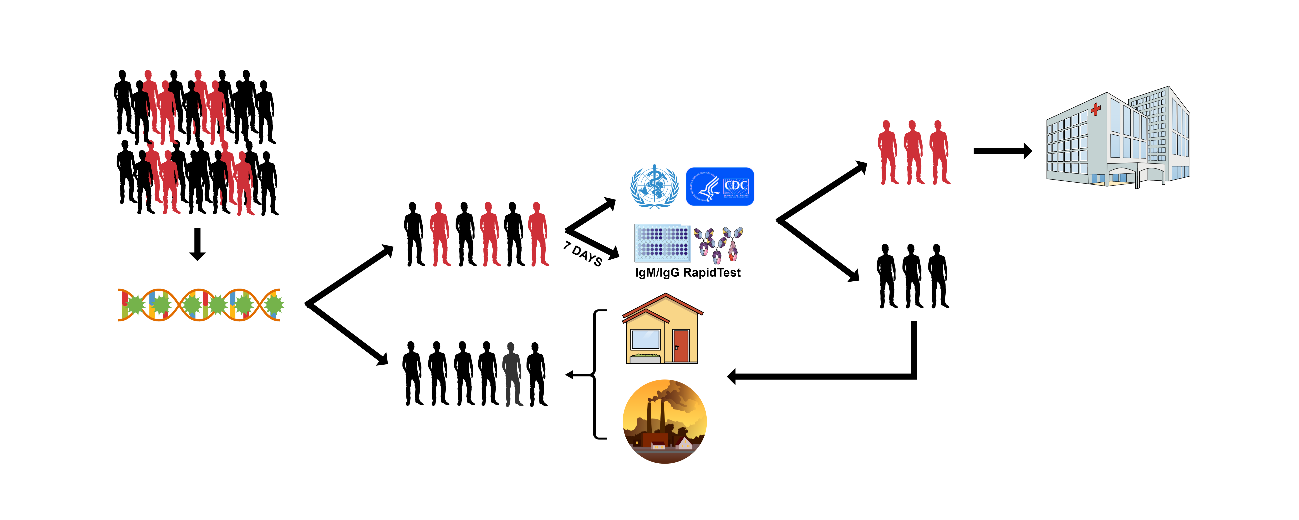


**Supplementary Figure S3**. Illustration of large-scale negative SARS-CoV-2 screening. Individuals from a given population can be screened through the SYBR molecular test presented in this study. All individuals who test negative can immediately resume their work routine (i.e., those belonging to essential work groups). Those who test positive should be tested according to WHO or CDC protocols or else wait in isolation for seven days to be diagnosed with a rapid test. For those who test positive according to WHO or CDC protocols or rapid testing, medical care and isolation should be applied according to disease severity. For those who test negative in the rapid test, social isolation with medical monitoring should continue only for 7 days, not the 14 days recommended for suspected cases. This image was created by authors using Mind the Graph (https://mindthegraph.com).


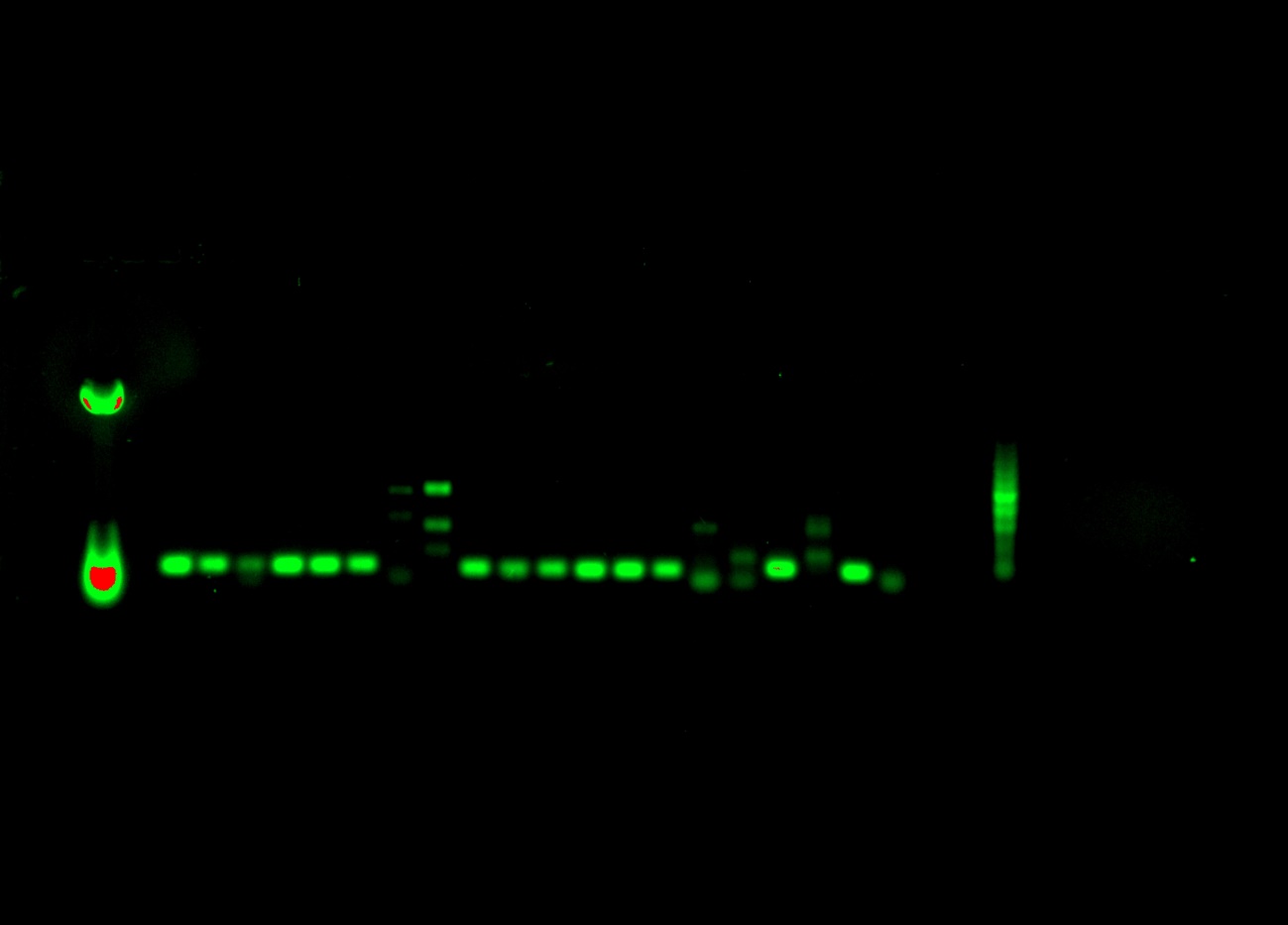


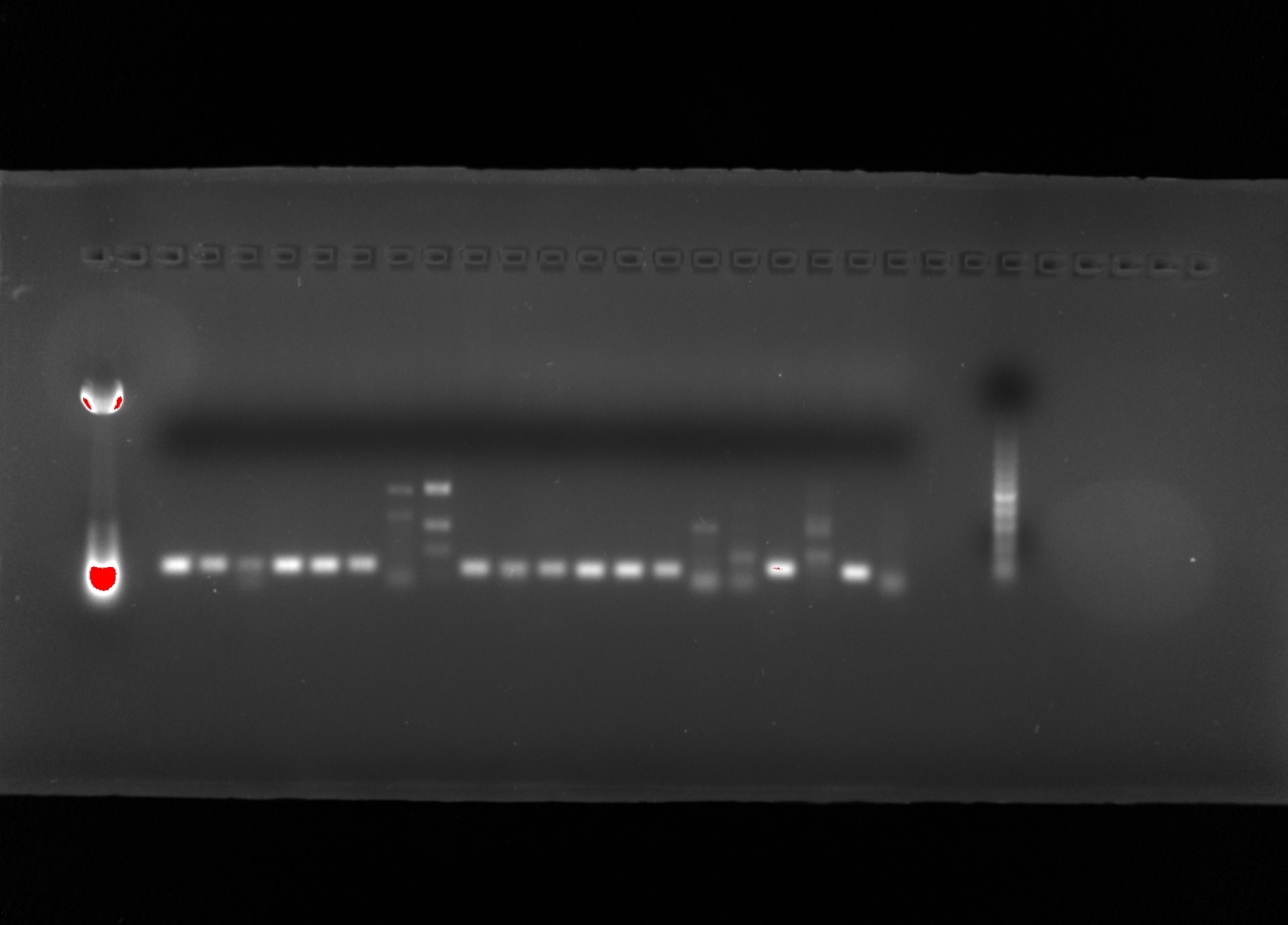
 **Supplementary Figure S4.** 2% agarose gel electrophoresis used in figure **4A** and **5C.**  ***FIGURE 4A***: Amplicons produced by RT-PCR without UDG activation. 110 pb - hCOVassay1 primer (lanes 3-10) and 102 pb – hCOVassay2 primer (lanes 11 - 18). ***FIGURE 5C***: Lane 19: amplicon of a SARS-CoV-2–positive sample obtained using the hCOVassay1 primer (111 bp). Lane 20: SARS-CoV-2–negative sample amplified using hCOVassay1 primer. Lane 21: SARS-CoV-2–positive sample amplified using the hCOVassay2 primer (102 bp). Lane 22: SARS-CoV-2–negative sample amplified using the hCOVassay2 primer.


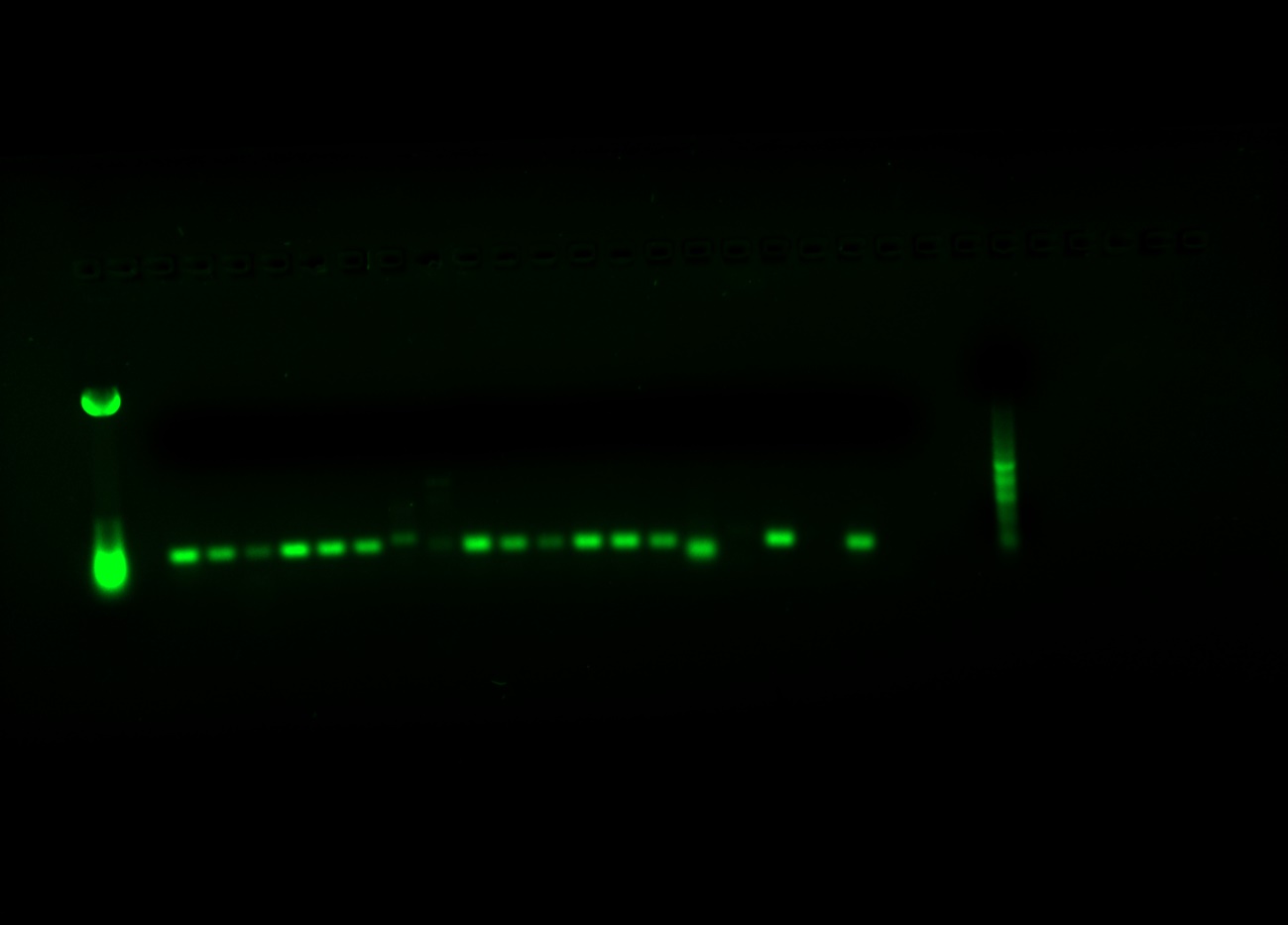


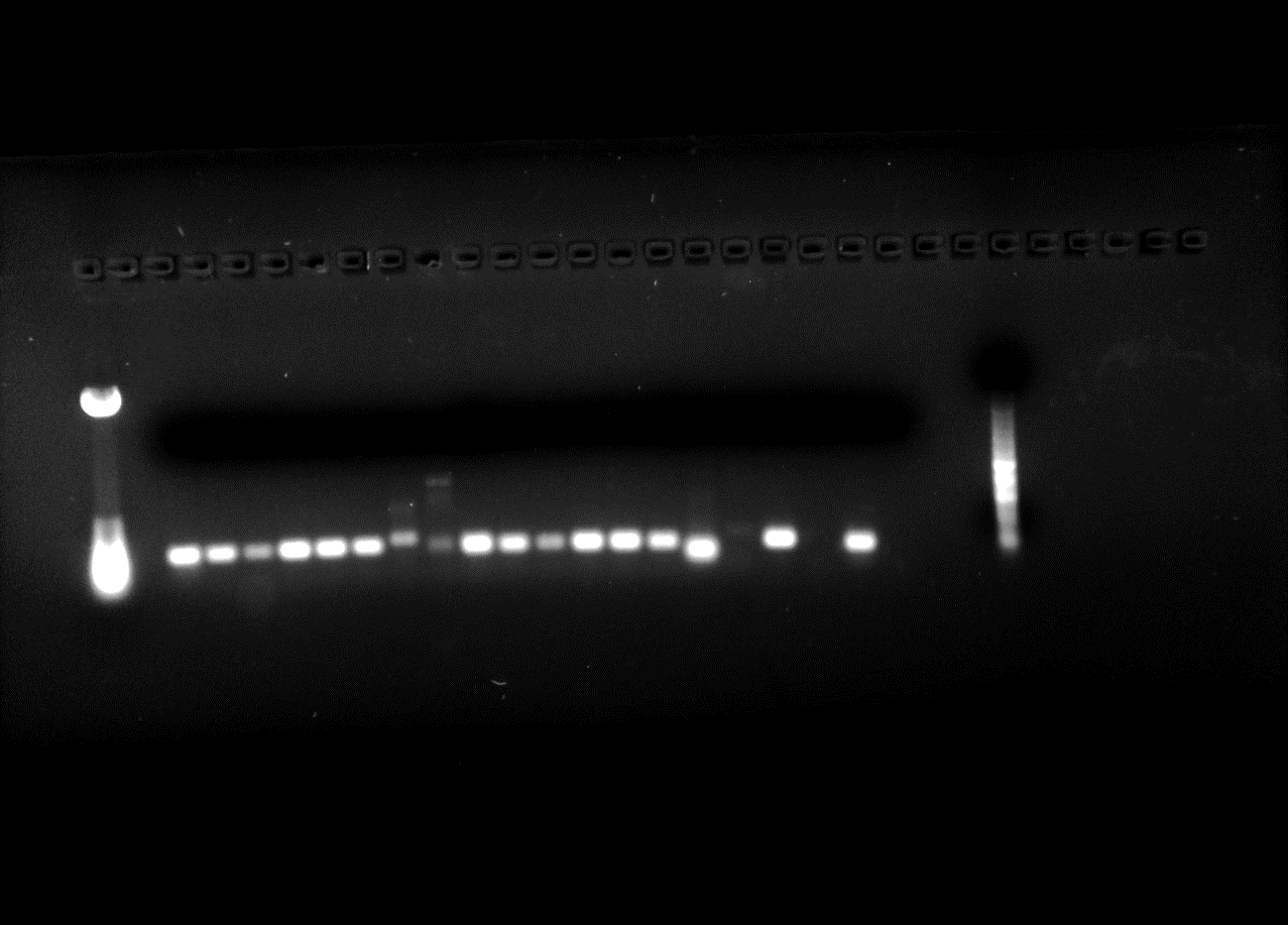


**Supplementary Figure S5.** 2% agarose gel electrophoresis used in figure 4B and 6C. ***FIGURE 4B*:** Amplicons produced by RT-PCR with UDG activation. 110 pb - hCOVassay1 primer (lanes 3-10) and 102 pb – hCOVassay2 primer (lanes 11 - 18). ***FIGURE 6C:*** Lane 19: amplicon of a SARS-CoV-2–positive sample using the hCOVassay1 primer set (111 bp). Lane 20: amplicon of a SARS-CoV-2–negative sample using the hCOVassay1 primer set (111 bp). Lane 21: amplicon of a SARS-CoV-2–positive sample using the hCOVassay2 primer (102 bp). Lane 22: amplicon of a SARS-CoV-2–negative sample using the hCOVassay2 primer (102 bp).


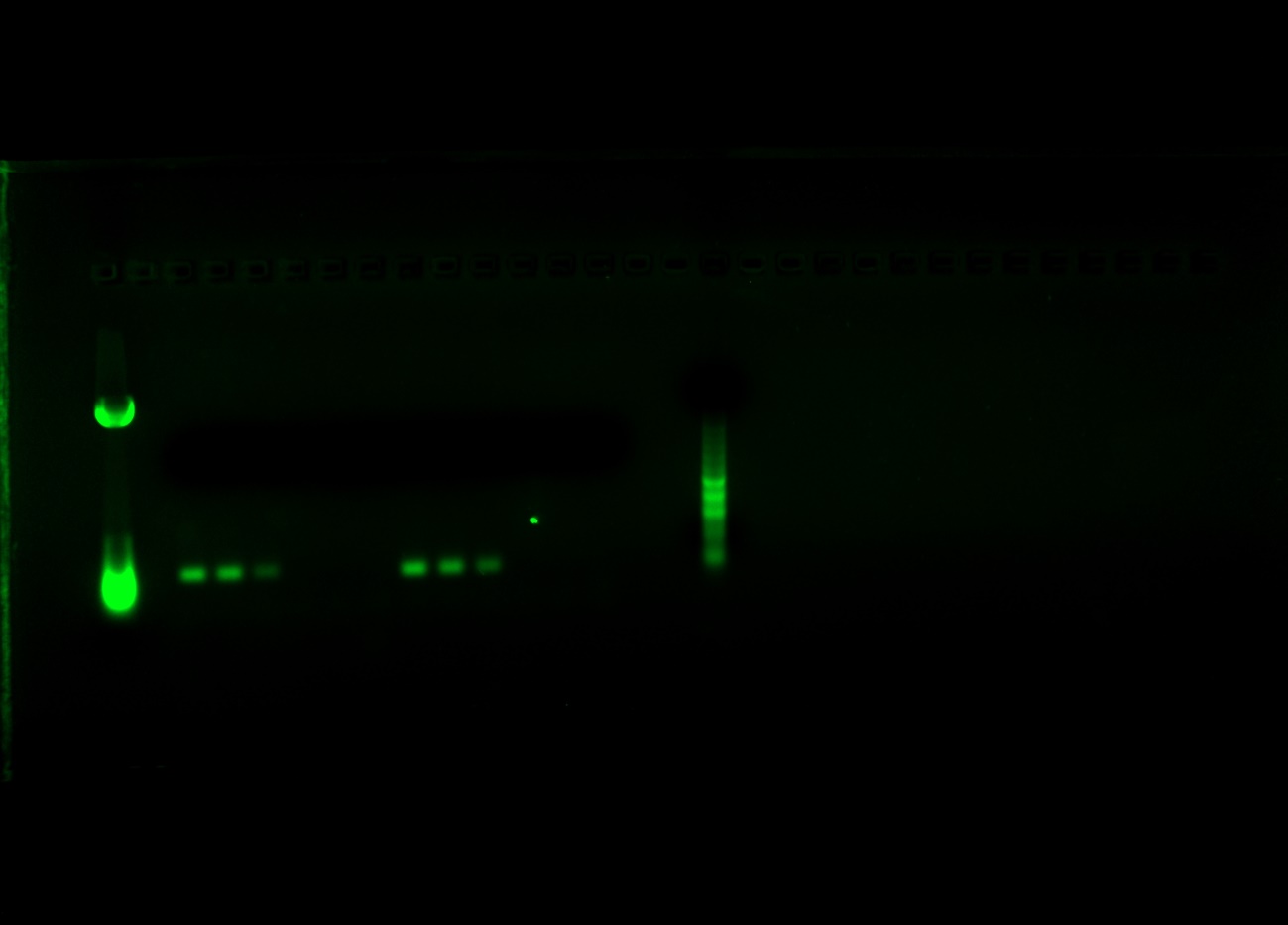


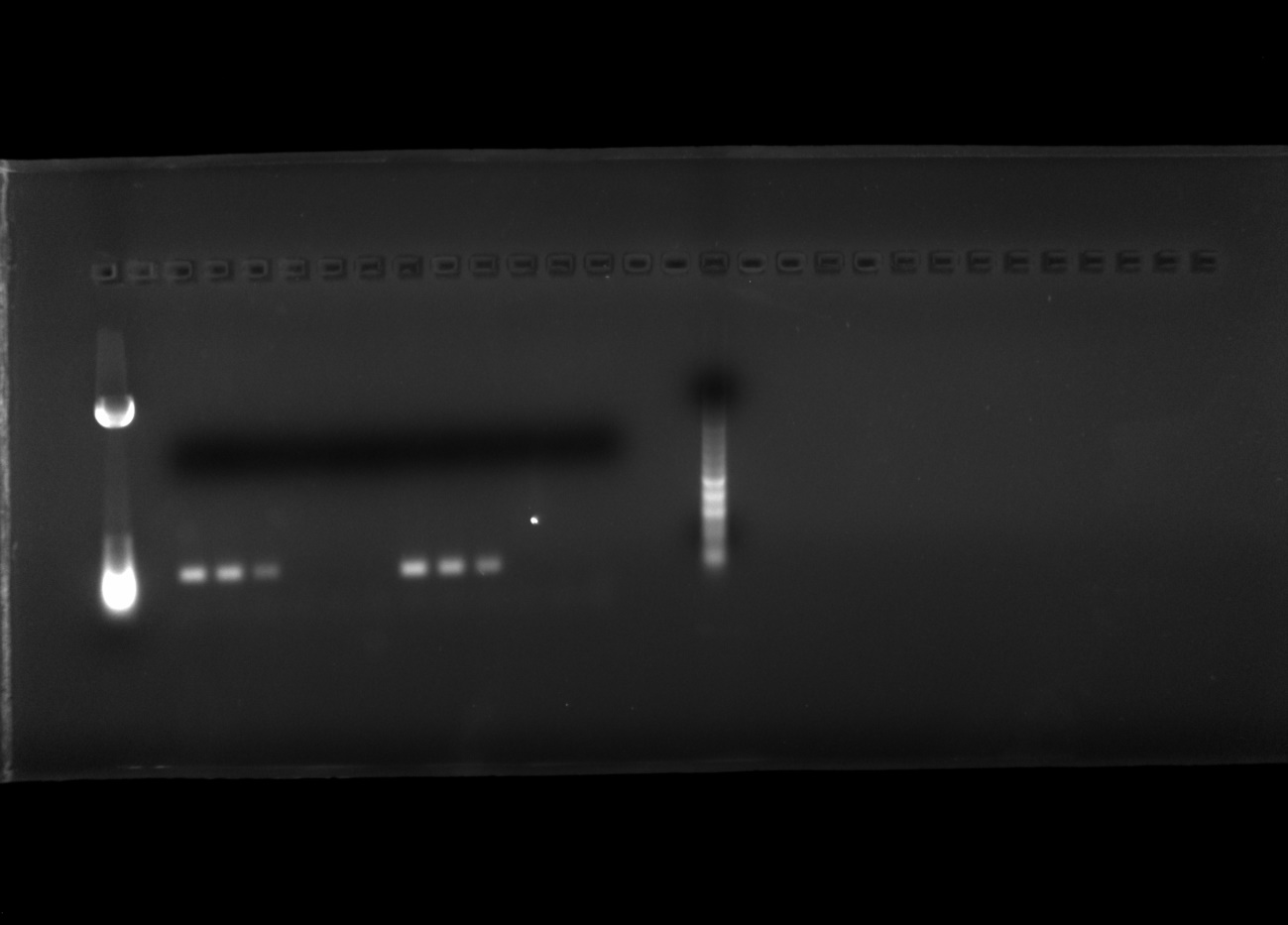


**Supplementary Figure S6.** 2% agarose gel electrophoresis used in ***FIGURE 6D***. Lanes 1, 2 and 3: amplicons of SARS-CoV-2–positive samples using hCOVassay1 primer dilutions of 100 ng, 50 ng, and 10 ng, respectively. Lanes 7, 8 and 9: amplicons of SARS-CoV-2–positive samples using hCOVassay2 primer at dilutions of 100 ng, 50 ng, and 10 ng, respectively. Lanes 4, 5, 6, 10, 11 and 12: amplicons of SARS-CoV-2–negative samples obtained using both pairs of primer**.**


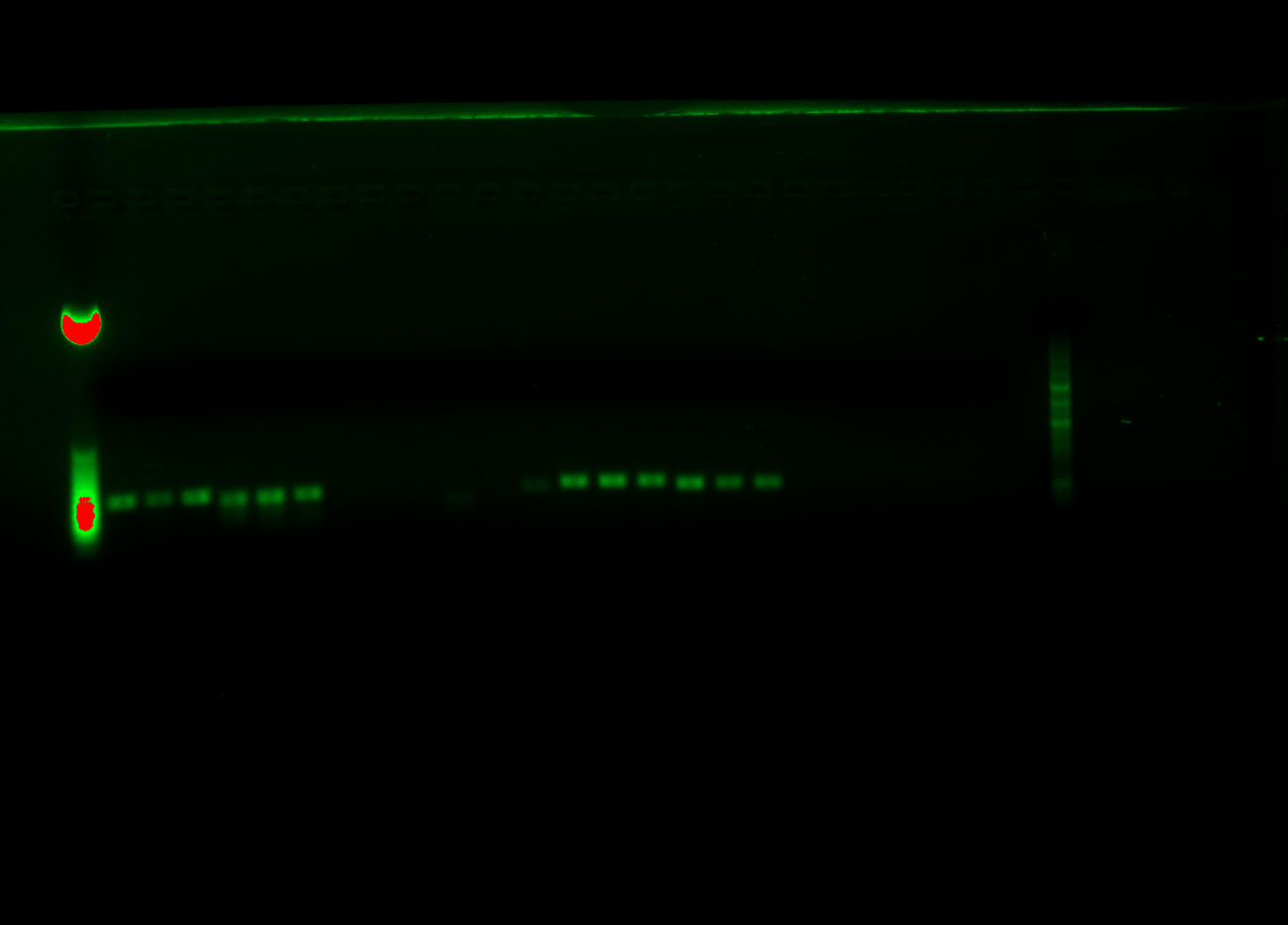


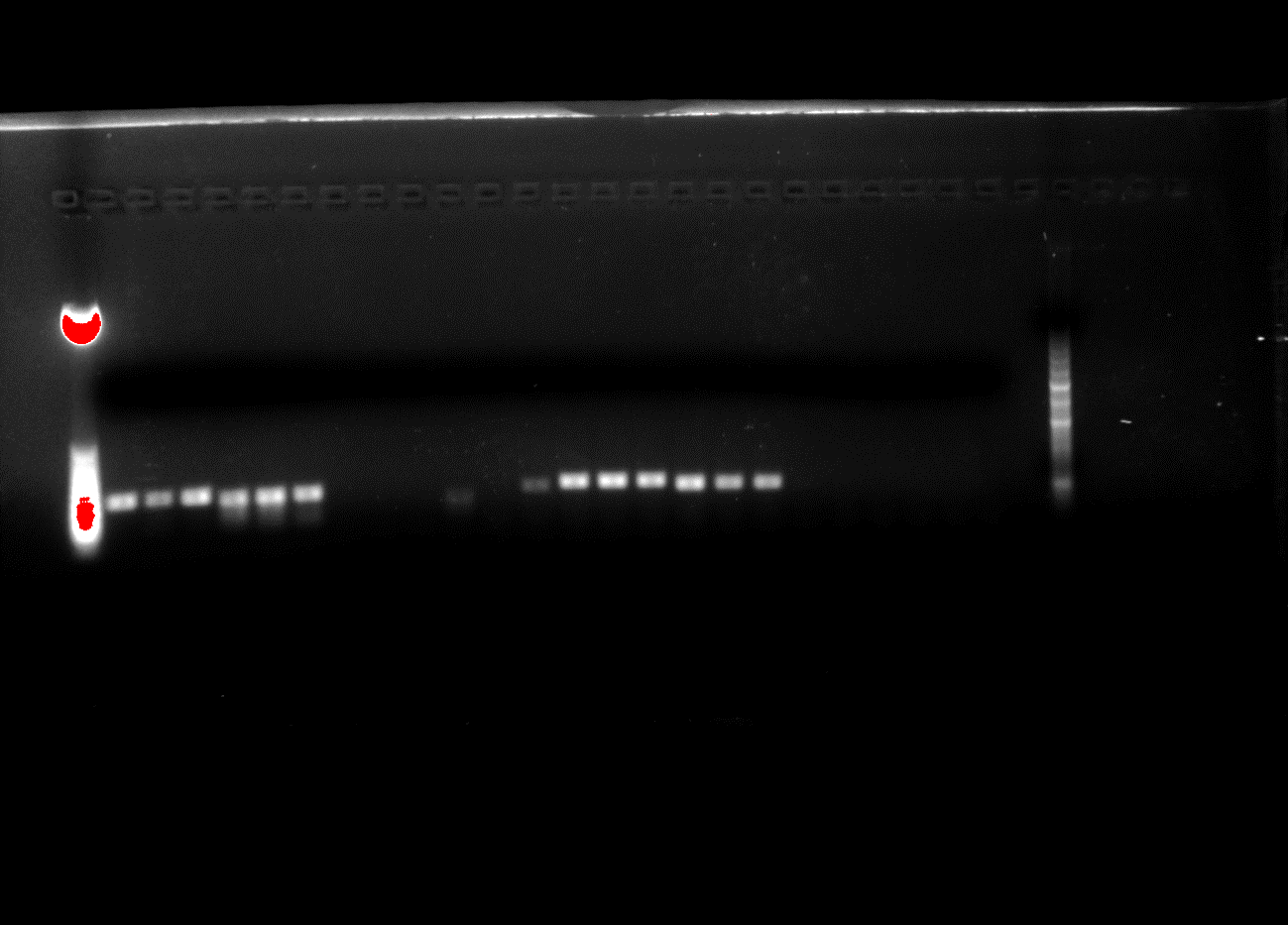


**Supplementary Figure S7.** 2% agarose gel electrophoresis used in ***FIGURE 8F*.** Electrophoresis of 3 samples and 3 controls for both tested primers. Lanes 14-16: hCOVassay1 primer. Lanes: 17-19: hCOVassay2 primer. Lanes 20-22 and 23-25: negative controls amplified using the hCOVassay1 and hCOVassay2 primer sets, respectively.
